# Geographic Variation in *Peromyscus leucopus* Population Size and Small Mammal Community Richness in Northern Pennsylvania

**DOI:** 10.1002/ece3.74535

**Published:** 2026-10-26

**Authors:** Michael G. Rowley, Gaimi Davies, Priscilla Langlais, Matthew Williams, Nicole Chinnici

**Affiliations:** ^1^ Dr. Jane Huffman Wildlife Genetics Institute East Stroudsburg University of Pennsylvania East Stroudsburg Pennsylvania USA

## Abstract

The white‐footed mouse, *Peromyscus leucopus* , is a generalist species found throughout the eastern and central United States that serves as an important reservoir for many tick‐borne pathogens relevant to human health. Patterns of *P. leucopus* relative abundance within small mammal communities have been shown to correlate with the prevalence of *Borrelia burgdorferi* , the main causative agent of Lyme disease, in vector species. However, efforts to investigate patterns of *P. leucopus* population size and small mammal community structure often lack geographic variation. In this study, we characterized patterns of *P. leucopus* population size and small mammal community species richness across a broad geographic range in Pennsylvania. Population sizes and small mammal community richness values in northeastern Pennsylvania (mean $\hat{N}$ = 28.6; mean richness = 5.3) were greater than those in northwestern Pennsylvania (mean $\hat{N}$ = 19.7; mean richness = 2.8). Projected changes in climate and the expansion of human‐caused disturbance have the potential to greatly influence the prevalence of *P. leucopus* in small mammal communities, so regular characterizations of *P. leucopus* community dynamics will likely be helpful to inform projections and assessments of human exposure to tick‐borne pathogen risk.

## Graphical abstract

The white‐footed mouse, *Peromyscus leucopus* , is a generalist species that serves as the primary reservoir for *Borrelia burgdorferi* , the causative agent of Lyme disease. Current estimates of *P. leucopus* population size in Pennsylvania lack geographic variation and context regarding the diversity of their communities. We present research describing differences in *P. leucopus* population size, small mammal species richness, and the relationships between these variables across a wide geographic area in northern Pennsylvania.

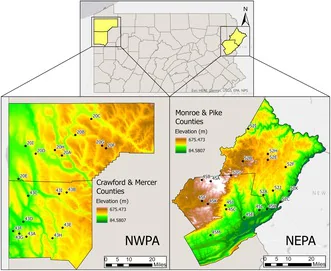

## 1 Introduction

Small mammals play key roles in forest ecosystems through granivory (Lacher et al. 2019), control of invasive pest populations (Ostfeld et al. 1996), and supporting carnivore populations (Lacher et al. 2019). Descriptions of small mammal community health through estimates of abundance and diversity can therefore be informative of overall ecosystem health. This has led to small mammals being considered indicator species of forest ecosystems (Pearce and Venier 2005; Avenant 2011; Ayodele 2025). Small mammal communities are also linked to human health through the retention and distribution of zoonotic diseases, most notably those caused by tick‐borne pathogens (Ostfeld et al. 2018). The species richness of small mammal communities and relative abundance of competent reservoir hosts has been related to the prevalence of pathogens in vector species (Ostfeld and Keesing 2000; Xavier et al. 2012). Similar to most other animal communities, small mammals are also responsive to long‐term changes in habitat structure, as seen with habitat fragmentation (Gomes et al. 2011; Mason et al. 2022) and urbanization (Chernousova et al. 2014; Gortat et al. 2014), as well as climate change (Moritz et al. 2008; Myers et al. 2009; Rowe and Terry 2014; Santoro et al. 2017; Lupone et al. 2024). Many small mammal species also exhibit cyclical population patterns relating to mast production; however, variation among these cycles and their drivers remain poorly understood (reviewed by Andreassen et al. 2021). Therefore, up‐to‐date profiles of small mammal communities can help inform best management practices aiming to mitigate human‐induced disturbances.

One species that has garnered particular attention is the white‐footed mouse, *Peromyscus leucopus* , a native generalist species (King 1968) with an expanding geographic range (Roy‐Dufresne et al. 2013; Garcia‐Elfring et al. 2017) that acts as the primary reservoir for *Borrelia burgdorferi* (Mather 1993), a causative agent of Lyme disease. As a generalist species particularly associated with disturbance (Linzey et al. 2012), habitat fragmentation (Wilder et al. 2005; Anderson and Meikle 2006), and climate change (Long 1996; Myers et al. 2009), *P. leucopus* ' distribution range is projected to continue expanding (Garcia‐Elfring et al. 2017). This will likely lead to changes in small mammal community composition, both due to its own introduction and due to its ecological replacement of other species, like the Eastern deer mouse, *Peromyscus maniculatus* (Long 1996; Wan 2014). *Peromyscus leucopus* has also been implicated in the spread of Lyme disease through its strong association with blacklegged ticks ( *Ixodes scapularis* ) (Bouchard et al. 2011; Millien et al. 2023; Dill et al. 2024). Relative to other hosts, *P. leucopus* is a highly competent reservoir for *B. burgdorferi* (Ostfeld et al. 2018); *P. leucopus* abundance has been directly linked to greater *B. burgdorferi* prevalence in *I. scapularis* (Keesing et al. 2009; Bouchard et al. 2011) and to general Lyme disease risk (Millien et al. 2023). Mitigation strategies targeting *P. leucopus* have been successful in reducing overall *B. burgdorferi* prevalence in *I. scapularis* (Stafford et al. 2020), further highlighting the importance of *P. leucopus* in regulating *B. burgdorferi* levels in the environment. Understanding the relative abundance of *P. leucopus* between communities and across regions can therefore help inform the deliberate planning of integrated pest management (IPM) strategies and/or regional assessments of tick‐borne pathogen infection risk. These patterns of abundance and community diversity are especially important to profile in Pennsylvania (PA), which has the fifth highest incidence rate of Lyme disease of any US state (2010–2023; CDC 2025). Populations of *I. scapularis* ticks and incidences of Lyme disease are historically well‐established in Eastern PA relative to those in Western PA (Larson et al. 2018; Eddens et al. 2019; Eisen and Eisen 2023; CDC 2025). Therefore, detailed profiles of underlying small mammal communities there may be especially pertinent to understanding mechanisms of tick‐borne disease expansion.

While recently published population size estimates for *P. leucopus* exist for limited geographic scopes in western (Linzey et al. 2012) and central PA (Goguen et al. 2015), there are no examples of population size estimates spanning multiple counties and geographic regions. Likewise, published examples of state‐wide descriptions of small mammal species richness patterns are rare (but see Joly and Myers 2001). However, given the recent westward expansion of Lyme disease in Pennsylvania (Eddens et al. 2019), as well as the potential for host species diversity and abundance to influence tick‐borne disease prevalence (Bouchard et al. 2013; Wang et al. 2023), geographically varied descriptions of host diversity and estimates of population sizes for *P. leucopus* may implicate human health. To determine whether *P. leucopus* population sizes or small mammal community richness influence rates of tick‐borne pathogen presence and/or human incidences of infection in PA, we need localized assessments of reservoir population size and species richness; we accomplished this by conducting live small mammal trapping across sites with varied habitats in northeastern and northwestern Pennsylvania. These data provide the first direct regional comparisons of *P. leucopus* population sizes and small mammal species richness patterns in Pennsylvania.

## 2 Methods

### 2.1 Field‐Site Description

Field work took place across 38 field sites located in Monroe, Pike (both in northeast Pennsylvania, hereafter “NEPA”), Crawford, and Mercer Counties (both in northwest Pennsylvania, hereafter “NWPA”) in northern Pennsylvania (Figure 1). Each county contained nine field sites; however, two sites (one in Monroe County and one in Pike County) were discontinued halfway through the study due to extremely low capture rates and were replaced with two new sites; discontinued sites were excluded from population size analyses. Field sites consisted of an even mix of public parks, residential communities, and schools (three each per county).

**FIGURE 1:**
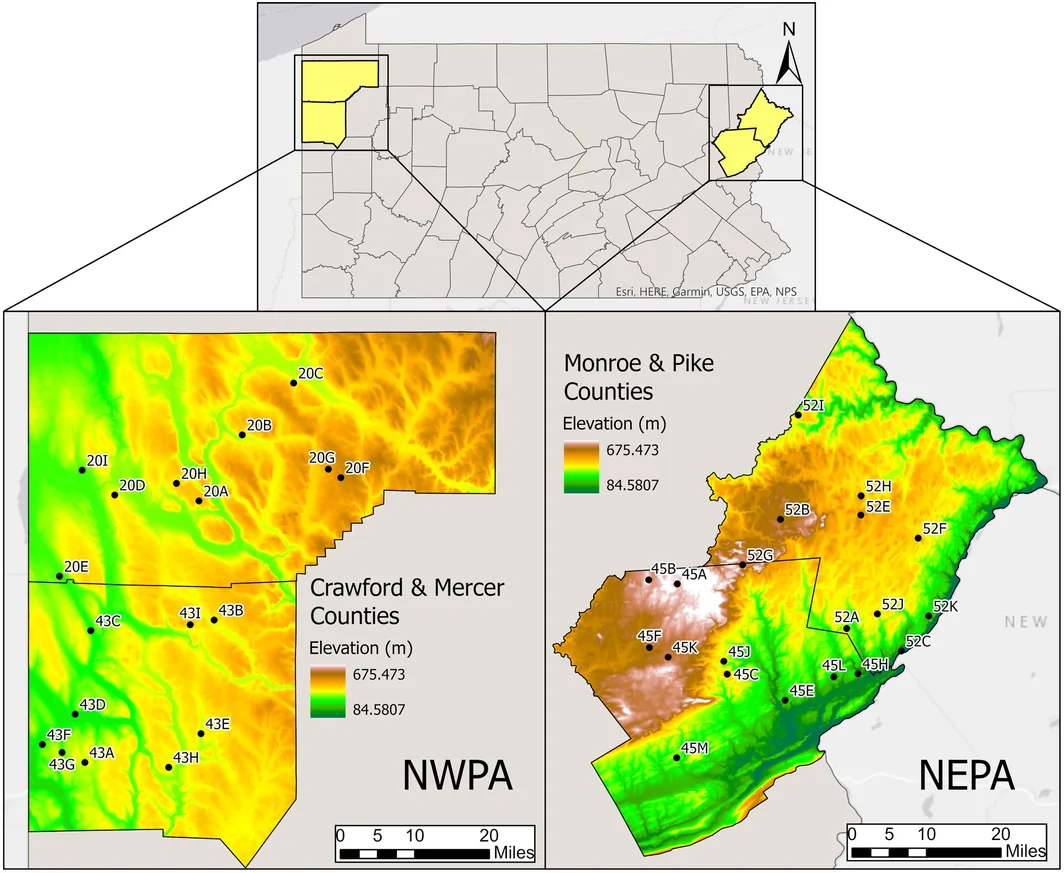
Map of Pennsylvania (top) with regional maps of NWPA (bottom left) and NEPA (bottom right) counties with elevation. Numerical values of sites refer to the county (20 = Crawford, 43 = Mercer, 45 = Monroe, and 52 = Pike County) while letters are used to represent each distinct site within counties.

Small mammal trapping was conducted along trails and forest edges following an 825 m‐long transect at each site. Sherman traps (H. B. Sherman Trap Inc., Tallahassee, FL, USA) were placed every 10 m along a parallel second transect (i.e., the “trap line”) located 10 m away from the trail/edge transect. Due to curves in the trails and forest edges, the trap lines varied in length, resulting in variable trap numbers per site (average = 83.9 traps/site; range = 73–91). Each trap was equipped with a ball of peanut butter‐oat mixture and polyester nesting material to provide thermal insulation. Trap locations were flagged to maintain consistent locations from season to season; traps were placed within 1 m of their respective flags for a maximum variation of around 2 m. Traps were placed flat to the ground and along logs or rocks (possible small mammal “highways”) if available close enough to their designated locations.

### 2.2 Small Mammal Trapping

*Peromyscus leucopus* and other small mammals were captured at each field site during its trapping week, defined by three consecutive trapping nights per site per trapping season (Fall 2022, Spring 2023, Summer 2023, Fall 2023, Summer 2024, Fall 2024, Summer 2025; see Appendix 1). A total of 2–6 sites were trapped per week across both regions, with trapping seasons taking place over an average of 48 days (range: 38–59). All small mammals were handled in accordance with approved East Stroudsburg University Institutional Animal Care and Use Committee (IACUC) protocols (IACUC protocol #ESU‐2022‐2). Newly captured small mammals were given a subcutaneous passive integrated transponder (Avid Identification Systems Inc., Norco, CA, USA; hereafter PIT tag) for future identification. Each captured individual was identified to species level to the best of the observer's ability; observers were trained to identify local small mammal species via morphological measurements (e.g., mass, tail/body length) and pelage, especially the differences between *Peromyscus* species. In situations where the morphology was unclear, the Peterson Field Guide to Mammals, 4th edition (Reid 2006) was used to clarify field identification. Small mammals that observers were unable to identify to species level due to the overlapping morphologies between *P. leucopus* and *P. maniculatus* were instead identified to genus level and excluded from species‐level richness analyses. Sex of mature *P. leucopus* was determined via examination of the anogenital length (Watts and Aslin 1981); individuals with unknown sex were excluded from statistical comparisons between sexes. Small mammals were released at their capture locations immediately after processing.

### 2.3 Statistical Analyses

*P. leucopus* population size estimates were generated using the robust design modeling approach (Kendall and Pollock 1992) with full likelihood and heterogeneity with parameters *pi*, *p*, and *c* (Kendall et al. 1997, 1995), available in program MARK version 11.1 (White and Burnham 1999). Under robust design, all captures are assumed to take place during discrete “capture events” separated by time intervals of known length (Schwarz and Arnason 1996); we defined each capture event as an individual trapping day (7 seasons with 3 nights each, resulting in 21 distinct events) with time intervals measured as the days elapsed between consecutive nights. Robust design models employ both open‐population (between seasons, i.e., “primary occasions”) and closed‐population (between days within a given season, i.e., “secondary occasions”) frameworks, enabling estimation of survival rate (*S*), abundance of nonobserved individuals (*f*_0_; used to derive total population size, $\hat{N}$), capture and recapture rates (*p* and *c*, respectively), temporary immigration and emigration (*γ*′ and *γ*″, respectively), and heterogeneity in parameters between discrete groups (like sex) in the population (*π*).

Since we were mostly interested in assessing differences in survival rate, population size, and sex ratio across time and space among our *P. leucopus* populations, we assembled an initial (univariate) model set that included variation in *S*, *f*_0_, and *π* across time, sex, and region (NEPA/NWPA). This amounts to nine possible combinations, minus the redundant combination of *π* varying between sexes, for a total of eight initial models (Table 1). Each of these eight models were compared (using AICc) against a null model, which itself was chosen from a comparison between two possible null models: one that was constrained such that *p = c* and one that estimated *c* and *p* separately. Additionally, to assess the best scope for assessing geographic variation, we chose well‐fitting regional‐variation models from the initial set and compared them with models assembled to vary by county instead; the best‐performing scope for geographic variation was then used for those initial models for future model selection processes. We were not interested in differences between immigration and emigration rates, so we constrained all models such that *γ*′ = *γ*″. As trapping was not conducted consistently at each site during each trapping event (e.g., no NWPA sites were trapped during Fall 2022 [events 1–3] and no NEPA sites except one were trapped during Fall 2023 [events 10–12]; see Appendix 1), we manually assigned a detection rate (*p*) value of 0 for such occasions across all fitted models. Following initial model comparison with AICc, we combined parameters from the best‐fitting univariate models to assemble secondary (multivariate) models, then repeated this process until we arrived at the best‐fitting model overall. We fit each model with multiple link functions (log, loglog, or logit) and proceeded with the best‐fitting version for model selection. The estimated parameter values from the best‐fitting model were then used to derive event‐ and site‐specific population size estimates and confidence intervals (Otis et al. 1978).

**TABLE 1:** List of null and initial models used to estimate *P. leucopus* population size. Only parameters that varied between fit models (*S*, *π*, and *f*_0_) are displayed.

Null models	Initial set
*S*(.) *π*(.) *f*_0_(.)	*S*(sex) *π*(.) *f*_0_(.)
*S*(.) *π*(.) *f*_0_(.) (*p = c*)	*S*(region) *π*(.) *f*_0_(.)
	*S*(time) *π*(.) *f*_0_(.)
	*S*(.) *π*(region) *f*_0_(.)
	*S*(.) *π*(time) *f*_0_(.)
	*S*(.) *π*(.) *f*_0_(sex)
	*S*(.) *π*(.) *f*_0_(region)
	*S*(.) *π*(.) *f*_0_(time)

To determine species richness values for each site, we counted the number of unique species identified per site (Magurran 1988). Individuals that we could not identify to species level were excluded from species richness analyses. Independent regional comparisons of species richness and *P. leucopus* population size were completed using two‐sample *t‐*tests. Comparisons between species richness and estimated *P. leucopus* population size were done using linear models (function “lm” from *R* package “lme4” version 1.1‐38; Bates et al. 2003).

## 3 Results

Across all seven trapping seasons, we recorded a total of 8298 total small mammal captures from 5250 unique individuals, including 3040 recaptures. The total number of capture opportunities (the number of trapping nights per site multiplied by the number of traps per site) was 56,168, amounting to an overall small mammal capture rate of 14.78%. For *P. leucopus* specifically, we recorded a total of 6873 captures (an overall capture rate of 12.24%) from 4337 unique individuals, including 2715 recaptures.

### 3.1 Species Richness

Thirteen species of small mammal were positively identified following capture in Sherman traps across all seasons and field sites (in order of capture rate relative to total captures): *Peromyscus leucopus* (89.55% of total captures), *Blarina brevicauda* (3.40%), *Clethrionomys gapperi* (2.62%), *P. maniculatus* (1.83%), *Tamias striatus* (1.51%), *Microtus pennsylvanicus* (0.34%), *Glaucomys volans* (0.31%), *Sorex cinereus* (0.15%), *Napaeozapus insignis* (0.13%), *Sorex fumeus* (0.06%), *Tamiasciurus hudsonicus* (0.04%), *Zapus hudsonius* (0.04%), and *Parascalops breweri* (0.02%) (Table 2).

**TABLE 2:** Small mammal species captured between Fall 2022 and Summer 2025 with species‐specific capture numbers and rates.

Scientific name	Common name	NEPA captures	NWPA captures	Total captures	% Total captures
*Peromyscus leucopus*	White‐footed mouse	2638	1631	4269	89.55
*Blarina brevicauda*	Northern short‐tailed shrew	44	118	162	3.40
*Clethrionomys gapperi*	Southern red‐backed vole	125	0	125	2.62
*Peromyscus maniculatus*	Eastern deer mouse	86	0	86	1.80
*Tamias striatus*	Eastern chipmunk	53	19	72	1.51
*Microtus pennsylvanicus*	Meadow vole	16	0	16	0.34
*Glaucomys volans*	Southern flying squirrel	14	1	15	0.31
*Sorex cinereus*	Masked shrew	5	2	7	0.15
*Napaeozapus insignis*	Woodland jumping mouse	6	0	6	0.13
*Sorex fumeus*	Smoky shrew	2	1	3	0.06
*Tamiasciurus hudsonicus*	American red squirrel	2	0	2	0.04
*Zapus hudsonius*	Meadow jumping mouse	2	0	2	0.04
*Parascalops breweri*	Hairy‐tailed mole	0	1	1	0.02
Total		2993	1774	4767	100

Species richness was significantly higher in NEPA sites (mean = 5.25; sample variance = 3.57) than in NWPA sites (mean = 2.83; sample variance = 0.73) (*t* = −5.16; two‐tail *p* ≪ 0.05) (Figure 2). Within regions, species richness did not differ significantly between constituent counties: Monroe and Pike counties did not differ significantly from one another (*t* = 0.34; two‐tail *p* = 0.73), and neither did Crawford and Mercer counties (*t =* −1.41; two‐tail *p* = 0.18) (Figure 3).

**FIGURE 2:**
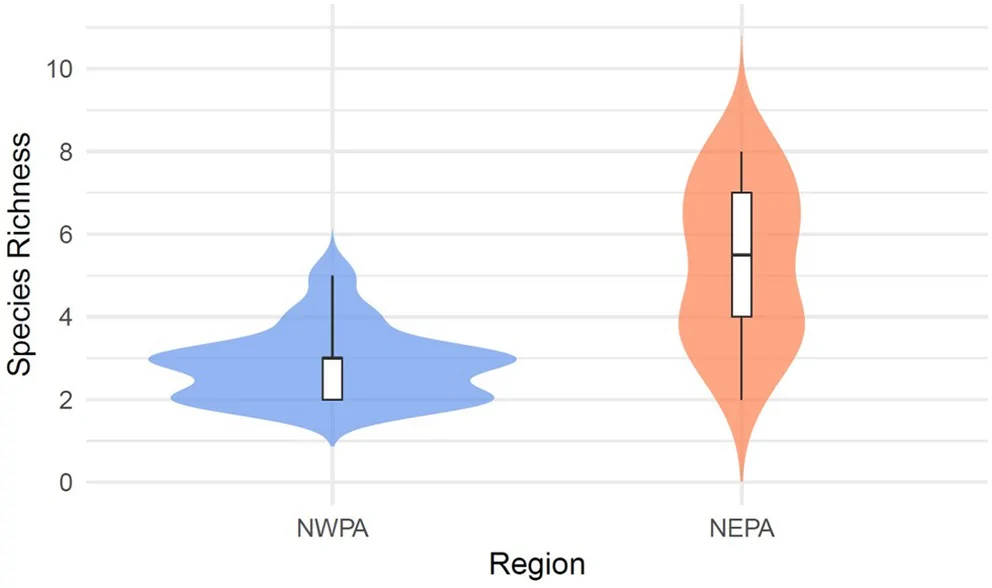
Species richness distributions grouped by region. Boxplots display interquartile range (box) and overall range (whiskers).

**FIGURE 3:**
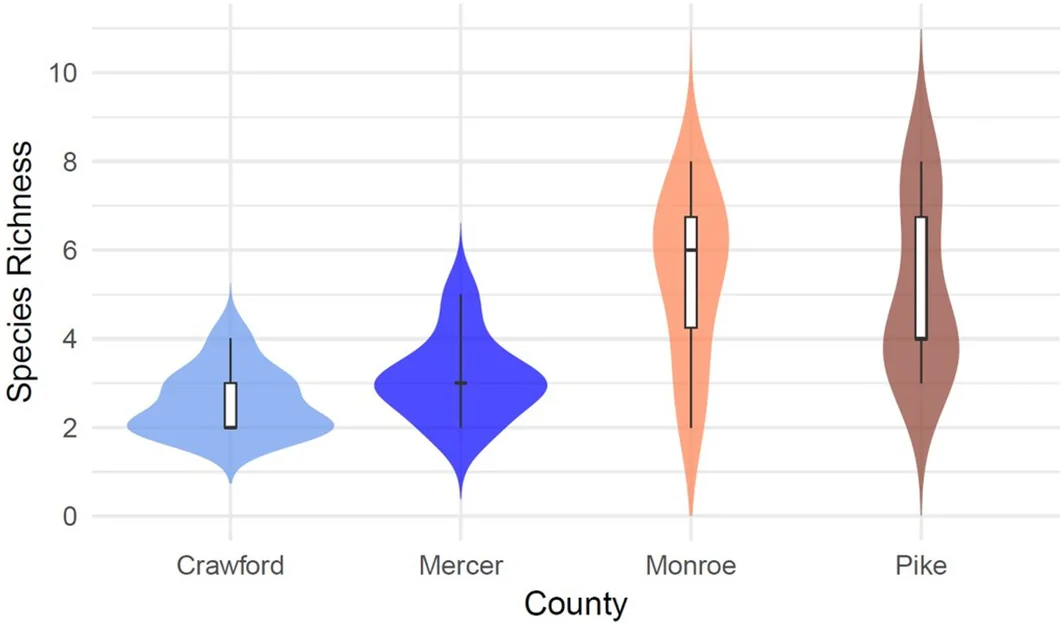
Species richness distributions grouped by county. Boxplots display interquartile range (box) and overall range (whiskers).

### 3.2 *Peromyscus leucopus* Population Sizes

Model selection led us to fit a candidate set of 23 models (Table 3), all but one of which achieved convergence. The model that failed to converge included county as a geographic covariate on *f*_0_, but the equivalent region‐varying model did converge. The best‐fitting null model estimated initial capture rates (*p*) separately from recapture rates (*c*), with *p* > c in all model estimates (excluding the *p* = *c* null model), so all other models were fit to estimate separate *p* and *c* values. Among the univariate models comparing geographic scopes (region and county), estimating regional variation generally resulted in slightly better‐fitting models (∆AIC < 5 from each other). The univariate model estimating regional abundance, however, greatly outperformed its county‐varying counterpart (∆AIC = 467.7).

**TABLE 3:** Final set of models, including those in the initial set and those created from combining variables from top‐performing candidate models.

Name	AICc	∆AICc	# Par.	Deviance	−2log(*L*)	Link
*S(time) π(.) f0(time + region)*	−31,348.58	0.00	31	−21,120.94	−31,410.73	Logit
*S(time) π(.) f0(sex + time)*	−31,208.95	141.64	31	−20,979.30	−31,269.09	Logit
*S(time) π(.) f0(sex + region)*	−30,958.25	390.33	14	−20,696.49	−30,986.28	Logit
*S(time) π(.) f0(sex + time + region)*	−30,937.44	411.14	38	−20,723.87	−31,013.66	Loglog
*S(time) π(.) f0(region)*	−30,925.31	423.27	12	−20,659.55	−30,949.34	Logit
*S(time) π(.) f0(.)*	−30,018.31	1330.28	10	−19,748.53	−30,038.32	Logit
*S(.) π(.) f0(time + region)*	−29,841.24	1507.34	26	−19,603.56	−29,893.35	Logit
*S(.) π(.) f0(sex + time + region)*	−29,838.19	1510.39	33	−19,614.57	−29,904.36	Log
*S(.) π(.) f0(sex + time)*	−29,623.71	1724.88	25	−19,384.01	−29,673.81	Logit
*S(.) π(.) f0(region)*	−29,274.71	2073.88	7	−18,998.92	−29,288.71	Logit
*S(.) π(.) f0(sex + region)*	−29,273.10	2075.48	8	−18,999.32	−29,289.11	Log
*S(.) π(.) f0(sex)*	−29,225.82	2122.76	7	−18,950.04	−29,239.83	Log
*S(.) π(.) f0(county)*	−28,807.05	2541.53	10	−18,537.27	−28,827.07	Logit
*S(.) π(.) f0(time)*	−28,223.47	3125.12	11	−17,955.69	−28,245.49	Log
*S(county) π(.) f0(.)*	−28,153.67	3194.91	9	−17,881.89	−28,171.68	Log
*S(sex) π(.) f0(.)*	−28,151.23	3197.35	8	−17,877.45	−28,167.24	Log
*S(region) π(.) f0(.)*	−28,149.32	3199.26	7	−17,873.54	−28,163.33	Log
*S(.) π(.) f0(.)*	−27,789.90	3558.69	6	−17,512.11	−27,801.90	Log
*S(.) π(region) f0(.)*	−27,785.89	3562.69	8	−17,512.11	−27,801.90	Logit
*S(.) π(county) f0(.)*	−27,781.89	3566.70	10	−17,512.11	−27,801.90	Logit
*S(.) π(time) f0(.)*	−27,779.88	3568.70	11	−17,512.11	−27,801.90	Log
*S(.) π(.) f0(.) (p = c)*	−26,826.09	4522.49	4	−16,544.30	−26,834.09	Logit

Out of the initial set of models (Table 1), those that estimated variation in *π* failed to outperform our chosen null model. Univariate models estimating variation in *S* all scored better than the null model, with the model estimating time‐specific variation in survival vastly outperforming the other two (∆AIC > 1860), which scored nearly identically to one another. All initial models that estimated variation in *f*_0_ returned lower AICc scores than the null model, performing better than all other univariate models except the time‐varying *S* model.

Of the combination models we created as part of our model construction process, the best‐performing models were those that estimated *S* as a function of time. Models that estimated *f*_0_ with different combinations of sex, time, and region generally scored better than those with only a single covariate on *f*_0_, with the best‐performing combination being time and region. No multivariate models included covariates on *π*. The overall best‐fitting model estimated survival as a function of time and estimated abundance as a function of both time and region. The top‐scoring model used a logit link function.

Site‐ and event‐specific population estimates (derived $\hat{N}$ values), averaged across all capture seasons (except Fall 2022 in NWPA, for which no estimates were produced), ranged from 7.93–45.97 (Figure 4; for more detailed estimates, see Appendix 2). Population sizes were significantly higher in NEPA (average = 28.63; SD = 10.10) than in NWPA (average = 19.68; SD = 7.81) (*t* = −3.07; *p* = 0.004). Population sizes did not differ significantly between counties located in the same region: Monroe (average = 24.37; SD = 10.01) and Pike Counties (average = 32.89; SD = 8.64) did not differ significantly (*t* = −2.03; *p* = 0.06), and neither did Crawford (average = 21.60; SD = 9.19) and Mercer Counties (average = 17.76; SD = 6.08) (*t* = 1.04; *p* = 0.31).

**FIGURE 4:**
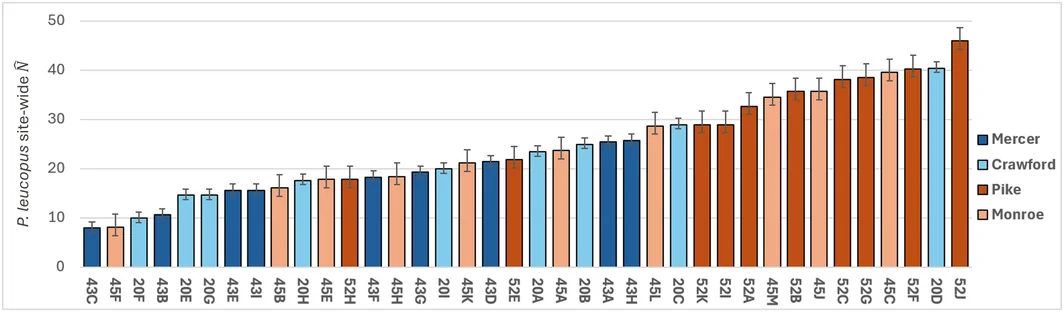
*Peromyscus leucopus* population size estimates ($\hat{N}$) averaged across all capture events and grouped by site. Error bars represent 95% confidence intervals.

Population size estimates varied qualitatively between years, with Summer estimates peaking in 2024 with a state‐wide average of 41.2 *P. leucopus* (Figure 5). Summers 2023 and 2025 exhibited state‐wide averages of 28.7 and 22.6 *P. leucopus* , respectively. State‐wide Fall estimates also peaked in 2024 with an average of 37.8 *P. leucopus* , followed by 2022 (20.7) and 2023 (17.0). The lowest average state‐wide population estimates were observed in Spring 2023 (9.2). Seasonal population estimates also qualitatively differed between regions (Figure 6), as NEPA had a single peak in Summer 2024, whereas NWPA estimates had two distinct peaks in Fall 2023 and Summer 2025.

**FIGURE 5:**
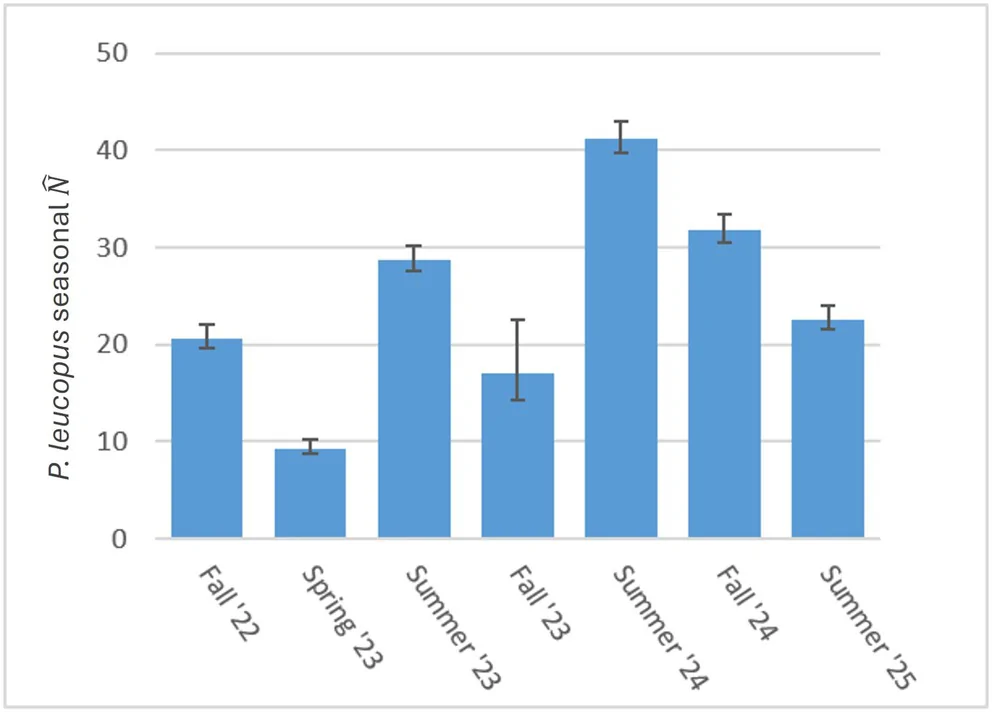
Seasonal state‐wide *P. leucopus* population size estimates ($\hat{N}$). Error bars represent 95% confidence intervals.

**FIGURE 6:**
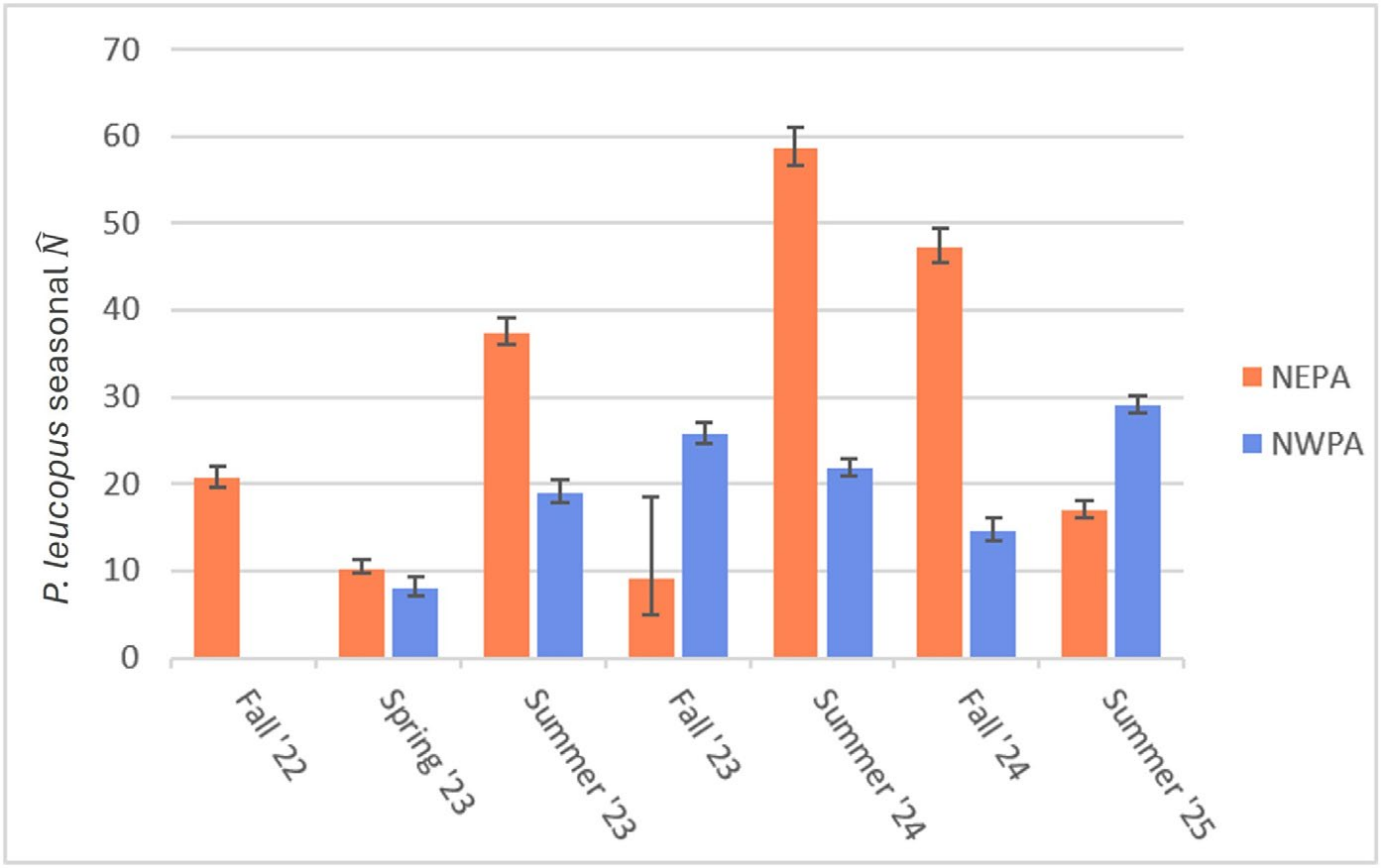
Seasonal region‐specific *P. leucopus* population size estimates ($\hat{N}$). Error bars represent 95% confidence intervals.

### 3.3 Comparing Species Richness With *P. leucopus* Population Sizes

*P. leucopus* population sizes did not vary significantly with respect to species richness when considering both regions together (estimate = 1.67 ± 0.83 [SE], *t‐*value = 2.01, *p =* 0.052; Figure 7). No significant relationship was also observed for NEPA (estimate = 0.88 ± 1.24, *t‐*value = 0.70, *p =* 0.49; Figure 8) and NWPA sites (estimate = −3.06 ± 2.15, *t‐*value = −1.43, *p =* 0.17; Figure 9) separately.

**FIGURE 7:**
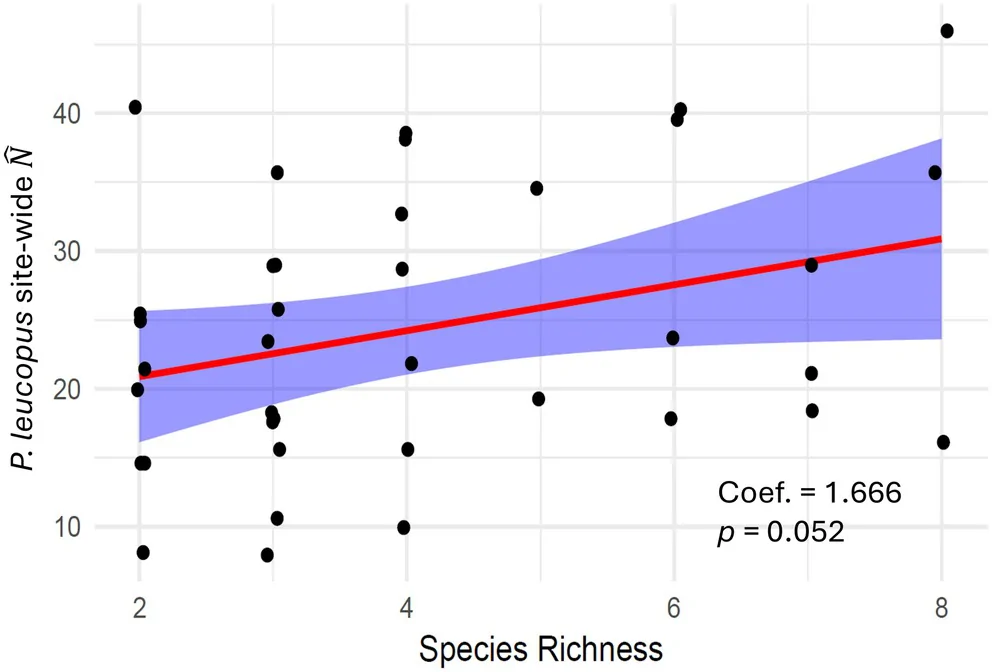
Results from a linear regression between *P. leucopus* estimated population sizes ($\hat{N}$) and small mammal species richness values across both NEPA and NWPA regions. Error band represents 95% confidence interval.

**FIGURE 8:**
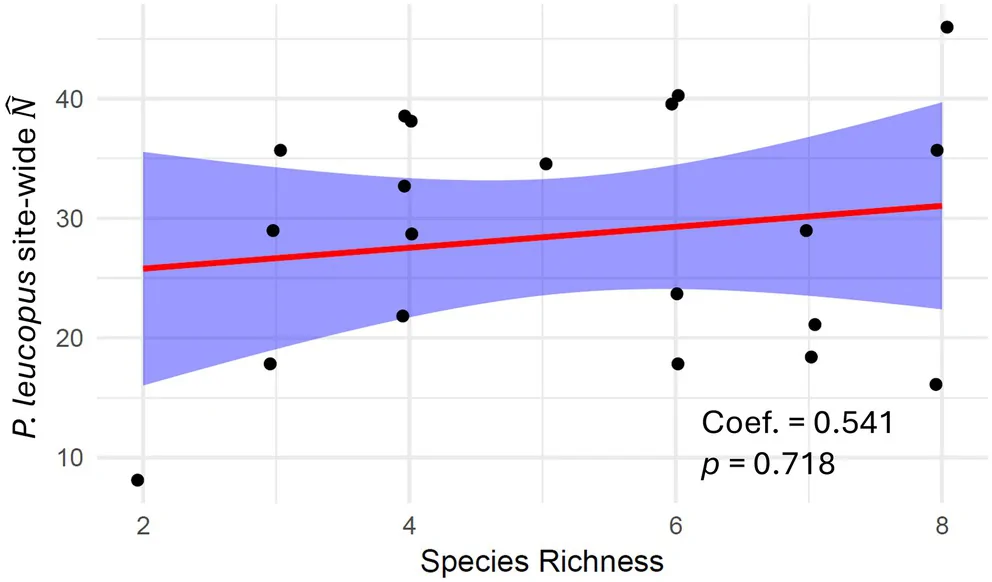
Results from linear regression between *P. leucopus* estimated population sizes ($\hat{N}$) and small mammal species richness across NEPA sites. Error band represents 95% confidence interval.

**FIGURE 9:**
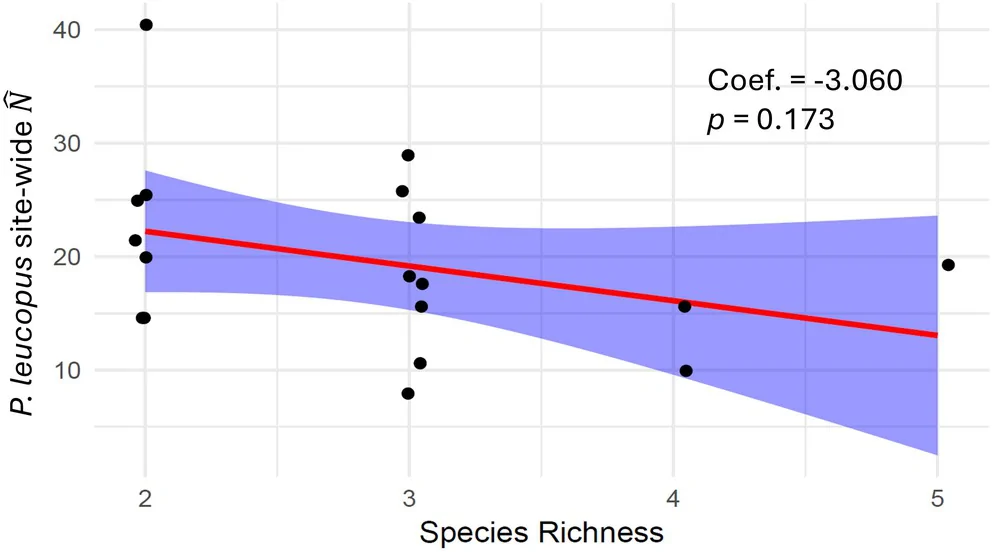
Results from linear regression between *P. leucopus* estimated population sizes ($\hat{N}$) and small mammal species richness across NWPA sites. Error band represents 95% confidence interval.

## 4 Discussion

### 4.1 Regional Variation in Small Mammal Species Richness

Small mammal species richness was significantly higher in NEPA than in NWPA but did not vary significantly within regions. Variation in small mammal species richness may be attributed to the physiographic area of our research sites. Sites across both of our study regions fall mostly within the Appalachian Plateau physiographic area, with parts of NEPA falling within the Ridge and Valley physiographic area (Sevon 2000). NWPA sites were located within the Eastern Broadleaf Forest (Oceanic) ecological province, while NEPA sites were mostly located within the Laurentian Mixed Forest ecological province (Bailey 1983). While the physiography of our regions was largely the same, the different ecological provinces they belonged to may have contributed to greater overall ecological diversity. A 2001 survey of terrestrial mammals (Joly and Myers 2001), while focusing on patterns of landscape feature association, reflected similar patterns between northeastern and northwestern areas in PA. This survey also concluded that the landscape feature with the strongest association with species richness was topographic variation (U.S. Geological Survey 1996). There was no direct correlation between species richness and elevation at our sites (via linear model; *F* = 2.76; *p* = 0.11), however, NEPA sites (average elevation = 407.6 m; SD = 143.1 m) exhibited much more topographic variation than NWPA sites (average elevation = 372.1 m; SD = 58.2 m) (via *F*‐test; *F =* 6.05; *p < <* 0.05) and therefore may provide more diverse landscapes, supporting the ecological needs of more species than NWPA sites. One notable example of a species found exclusively at NEPA sites was *P. maniculatus* , which occurred in 15 out of 20 NEPA sites (75%) and none of the NWPA sites, although three of the remaining five NEPA sites were only partially sampled (45F, 52E, 52H; see Appendix 1). While not as well‐studied as *P. leucopus* , *P. maniculatus* occurrence may influence rates of tick‐borne pathogen presence, as infection rates with *Borrelia burgdorferi* have been found to be comparable (Dill et al. 2024) or higher (Larson et al. 2021) in *P. maniculatus* than *P. leucopus* .

Lower richness values in NWPA could also have partially resulted from qualitatively higher rates of trap disturbance by mesocarnivores (mainly raccoons), which effectively reduced the sampling effort. To mitigate these effects while maintaining small mammal capture rates, we deployed raccoon exclusion devices (fixed sections of corrugated plastic tubing; see Roden‐Reynolds et al. 2018 for more details) at several sites where disturbance rates appeared high. Additionally, due to our limited study design (trapping along trails and edges for roughly 750 m), we likely are underestimating the true species richness values of small mammal communities, especially those that are associated with contiguous forest. Future research should plan to prevent or account for these changes in capture opportunity, especially in locations where foraging animals are likely to occur.

### 4.2 Regional Variation in *P. leucopus* Population Sizes

*P. leucopus* population sizes were higher at NEPA field sites than those in NWPA but did not vary significantly within regions. NWPA's proximity to Lake Erie (with Crawford County being closer than Mercer County) and/or its membership in the Eastern Broadleaf Forest ecological province (Bailey 1983) may promote differences in the ecological drivers influencing *P. leucopus* population size relative to NEPA. While the aforementioned patterns of physiography, topography, and ecology between the survey regions may have contributed to higher relative abundance of *P. leucopus* in small mammal communities, further investigation is needed to describe detailed habitat associations throughout PA. Research on *P. leucopus* habitat associations so far has shown that they thrive in human‐disturbed habitats (Linzey et al. 2012), prefer forests with complex understory (Anderson and Meikle 2006), and congregate in “edge” habitat (in small forest patches) (Anderson et al. 2003). As a generalist species (King 1968), *P. leucopus* may persist in conditions that are intolerable to other species, leading to their higher relative abundance in complex/disturbed habitats. Therefore, the next steps for research into patterns of *P. leucopus* abundance should involve comparisons with detailed measures of vegetation structure in addition to other biotic and abiotic ecological variables. Our research, part of a long‐term project monitoring small mammal communities and their impacts on the enzootic cycle for tick‐borne pathogens, aims to assess these potential habitat associations with *P. leucopus* population dynamics across geographic regions in PA.

The best‐performing model estimated survival rate (*S*) with respect to time, abundance of nonobserved individuals (*f*_0_) with respect to time and region, and initial capture rates (*p*) separately from recapture rates (*c*); initial *P. leucopus* capture rates were greater than recapture rates (*p =* 0.407; *c* = 0.275). Lower recapture rates were likely influenced by increased rates of trap disturbance over the course of a trapping bout (P. Langlais, personal observation), reducing our effective sampling efforts across successive trap nights. Our limited number of trap nights per season could also have disproportionately influenced recapture rates due to reduced opportunities to recapture mice that were first captured early in the trapping bout. The reduced capture rate following initial capture may also indicate a “trap‐shy” behavioral response to capture. Such a response may result from processing‐related stress, as well as emigration (death and/or movement from the area) within primary occasions (seasons), which our robust design approach assumes to be nonexistent. “Trap‐shyness” is relatively uncommon among *P. leucopus* trapping efforts, as well as *Peromyscus* mice generally (Hammond and Anthony 2006), but does not necessarily indicate that our trapping efforts are overly invasive. Rather, this difference in capture rates highlights the importance of accounting for potential behavioral responses to capture in animal population studies, as our abundance estimates may have been inflated had we relied on a single (likely higher) capture rate estimate.

Seasonal variation in *S* and *f*_0_ could likely be attributed to the cyclical pattern of rodent populations (reviewed by Andreassen et al. 2021); *P. leucopus* abundance cycles in particular have been tied to acorn crops in eastern deciduous forests (Wolff 1996; Elias et al. 2004; Ostfeld et al. 2018). Our 3 years of surveying *P. leucopus* populations has not been enough to observe the average period of a full population cycle (4 years; Elias et al. 2004), but we did observe qualitative fluctuations in seasonally averaged $\hat{N}$ values. These fluctuations, likely driven by cyclical patterns of seed production, are likely to cause ripple effects throughout the ecosystem, including pathogen prevalence among *I. scapularis* ticks (Keesing et al. 2009) and the abundance of prey arthropods that themselves influence oak health (Ostfeld et al. 1996; Jones et al. 1998). While oak trees are not ubiquitous at our sites (G. Davies, P. Langlais, personal observations), surveys of annual acorn crops across both regions may provide essential context for understanding drivers of regional differences in *P. leucopus* population cycles.

The population size estimates we present here are valuable for identifying patterns across time and for drawing comparisons between sites; however, their comparative value with other systems is limited. Our small mammal communities are characterized by open populations, so it is difficult to determine the spatial bounds of small mammal movement into and out of the trapping area. Estimates of small mammal density (individuals per unit area) would enable comparisons across other systems with different site layouts and trapping regimes; future attempts to identify large‐scale patterns of population‐ and community‐level change would benefit from a focus on producing generalizable metrics like density. While these limitations are present in our study, the data herein provide novel and detailed information on the structure of a tick‐borne pathogen reservoir community.

### 4.3 No Association Between Richness and *P. leucopus* Population Size

*P. leucopus* population sizes did not vary significantly with differences in small mammal species richness regardless of geographic scope (coef = 1.67, *p* = 0.052). Bouchard et al. (2013) observed a similar positive correlation but significant relationship between *P. leucopus* population sizes and small mammal richness in southeastern Canada. This observed relationship by Bouchard et al. (2013) is notably counterintuitive due to our current understanding of *P. leucopus* as generalist species (Wilder et al. 2005; Linzey et al. 2012) that thrive in disturbed habitats and the related potential for competitive exclusion (Wan 2014). Expanding the scope of our study to include more sites and/or additional seasons (trapping efforts remain ongoing) may help clarify whether this nonsignificant trend suggests an underlying biological process driving *P. leucopus* associations with high‐richness small mammal communities. Capture rates may also be influenced by different sources of trap disruption between regions: NWPA noted more raccoon‐related trap disturbance whereas NEPA had more black bear‐related disturbance. This reinforces the importance for future research into small mammal community dynamics to prioritize geographic sampling variation and measurement of ecological features.

### 4.4 Implications for Human Health

We observed *P. leucopus* at every site, so its presence alone in the community cannot be used to predict *I. scapularis* host availability. Instead, species diversity within the small mammal community, as well as the relative abundance of *P. leucopus* compared to other species within those communities would offer greater insights into variations of suitable hosts and vector reservoirs available. *B. burgdorferi* prevalence has been linked to the “dilution effect” theory which predicts a positive association between pathogen prevalence and the proportion of competent reservoir species (e.g., *P. leucopus* ) that comprise the available host pool (i.e., species richness) (Long 1996; Keesing et al. 2009, 2010; Millien et al. 2023). While we were unable to produce site‐specific population size estimates for every captured species, the lack of a statistically significant association between small mammal species richness and *P. leucopus* population size suggests that *P. leucopus* abundance is independent from the composition of their host community; assessment of *I. scapularis* tick density and *B. burgdorferi* prevalence is necessary to determine if changes to host community membership confer a dilution effect here. Higher species richness in a community can also lead to a greater chance of disease emergence (Keesing et al. 2010), so the impacts of changing community structures on human health are not straightforward. However, as the number one reported zoonotic disease in the US (Adrion et al. 2015), Lyme disease presents a particularly high risk to human health, warranting further investigation into patterns of occurrence and abundance of *P. leucopus* , its most competent reservoir.

## Author Contributions

**Michael G. Rowley:** conceptualization (supporting), data curation (lead), formal analysis (lead), investigation (supporting), methodology (lead), visualization (lead), writing – original draft (lead), writing – review and editing (equal). **Gaimi Davies:** conceptualization (supporting), data curation (supporting), investigation (equal), methodology (equal), project administration (supporting), supervision (supporting), validation (equal), writing – review and editing (supporting). **Priscilla Langlais:** conceptualization (supporting), data curation (supporting), investigation (equal), methodology (equal), project administration (supporting), supervision (supporting), validation (equal), writing – review and editing (supporting). **Matthew Williams:** conceptualization (supporting), data curation (supporting), investigation (equal), methodology (equal), project administration (supporting), supervision (supporting), validation (supporting), writing – review and editing (supporting). **Nicole Chinnici:** conceptualization (lead), funding acquisition (lead), investigation (lead), methodology (supporting), project administration (lead), resources (lead), supervision (lead), writing – original draft (supporting), writing – review and editing (lead).

## Funding

This work was supported by the Pennsylvania Department of Education (no. 4100092637) and the Pennsylvania Department of Health (nos. 4100097633 and 4100099815). The Pennsylvania Department of Education and the Pennsylvania Department of Health specifically disclaims responsibility for any analyses, interpretations, or conclusions.

## Conflicts of Interest

The authors declare no conflicts of interest.

## Data Availability

The data that support the findings of this study are openly available in Dryad at https://doi.org/10.5061/dryad.wh70rxx3d.

## Appendix 1 Field Sites

**Table:**

Site code	Town	County	Latitude	Longitude	Elevation (m)
45A	Tobyhanna	Monroe	41.213902	−75.407719	613
45B	Gouldsboro	Monroe	41.219947	−75.463368	619
45C	Swiftwater	Monroe	41.081964	−75.311009	386
45E	Arlington Heights	Monroe	41.043745	−75.198456	226
45F	Pocono Pines	Monroe	41.118595	−75.459895	552
45H	Saw Creek	Monroe	41.084733	−75.057227	250
45 J	Swiftwater	Monroe	41.100722	−75.317631	378
45 K	Pocono Pines	Monroe	41.106969	−75.425686	560
45 L	Saw Creek	Monroe	41.078087	−75.103798	248
45 M	Effort	Monroe	40.959558	−75.409310	333
52A	Saw Creek	Pike	41.150311	−75.075288	354
52B	Greentown	Pike	41.308234	−75.207584	554
52C	Pine Ridge	Pike	41.116009	−74.972857	111
52E	Lords Valley	Pike	41.314230	−75.053020	454
52F	Dingmans Ferry	Pike	41.280570	−74.940032	419
52G	Cresco	Pike	41.241631	−75.280938	574
52H	Lords Valley	Pike	41.342404	−75.050883	459
52I	Hawley	Pike	41.460072	−75.173105	402
52 J	Dingmans Ferry	Pike	41.170169	−75.019451	404
52 K	Dingmans Ferry	Pike	41.166988	−74.919599	256
20A	Meadville	Crawford	41.606532	−80.188453	410
20B	Saegertown	Crawford	41.702206	−80.104176	376
20C	Andersons Corner	Crawford	41.777420	−80.004500	384
20D	Conneaut Lake	Crawford	41.615187	−80.351767	393
20E	Jamestown	Crawford	41.497129	−80.459003	314
20F	Townville	Crawford	41.640320	−79.912750	505
20G	Guys Mills	Crawford	41.652590	−79.936920	468
20H	Meadville	Crawford	41.632037	−80.231792	414
20I	Linesville	Crawford	41.650974	−80.415251	326
43A	Hermitage	Mercer	41.225739	−80.409961	363
43B	Sandy Lake	Mercer	41.433420	−80.158950	374
43C	Greenville	Mercer	41.417881	−80.398147	305
43D	Transfer	Mercer	41.296084	−80.428702	277
43E	Mercer	Mercer	41.267773	−80.184486	363
43F	Sharon	Mercer	41.251875	−80.492282	312
43G	Hermitage	Mercer	41.240324	−80.454340	323
43H	Mercer	Mercer	41.218755	−80.247243	378
43I	Hadley	Mercer	41.426626	−80.205266	412

Site code	F '22	Sp '23	Su '23	F '23	Su '24	F '24	Su '25
45A	X	X	X		X	X	X
45B	X	X	X		X	X	X
45C				X	X	X	X
45E	X	X	X		X	X	X
45F	X	X	X				
45H	X	X	X		X	X	X
45J	X	X	X		X	X	X
45K	X	X	X		X	X	X
45L	X	X	X		X	X	X
45M	X	X	X		X	X	X
52A	X	X	X		X	X	X
52B	X	X	X		X	X	X
52C	X	X	X		X	X	X
52E	X	X	X				
52F	X	X	X		X	X	X
52G	X	X	X		X	X	X
52H					X	X	X
52I	X	X	X		X	X	X
52J	X	X	X		X	X	X
52K	X	X	X		X	X	X
20A		X	X	X	X	X	X
20B		X	X	X	X	X	X
20C		X	X	X	X	X	X
20D		X	X	X	X	X	X
20E		X	X	X	X	X	X
20F		X	X	X	X	X	X
20G		X	X	X	X	X	X
20H		X	X	X	X	X	X
20I		X	X	X	X	X	X
43A		X	X	X	X	X	X
43B		X	X	X	X	X	X
43C		X	X	X	X	X	X
43D		X	X	X	X	X	X
43E		X	X	X	X	X	X
43F		X	X	X	X	X	X
43G		X	X	X	X	X	X
43H		X	X	X	X	X	X
43I		X	X	X	X	X	X

## Appendix 2 Model Parameter Estimates

**Table:**

Site	Event	$\hat{N}$	95% CI‐lower	95% CI‐upper
45A	1	15.795601	14.794961	17.154487
45A	2	1.4445246	0.8455585	2.4677791
45A	3	15.627534	14.231757	17.39569
45A	4	7.6463315	3.4557572	16.918545
45A	5	51.031278	49.059462	53.3896
45A	6	64.457356	62.750627	66.539915
45A	7	9.7973828	8.9468446	11.019505
45B	1	13.795601	12.794961	15.154487
45B	2	2.4445246	1.8455585	3.4677791
45B	3	10.627534	9.2317573	12.39569
45B	4	7.6463315	3.4557572	16.918545
45B	5	31.031278	29.059462	33.3896
45B	6	37.457356	35.750627	39.539915
45B	7	9.7973828	8.9468446	11.019505
45C	1	3.795601	2.7949605	5.1544866
45C	2	1.4445246	0.8455585	2.4677791
45C	3	6.6275344	5.2317573	8.3956899
45C	4	38.646332	34.455757	47.918545
45C	5	114.03128	112.05946	116.3896
45C	6	62.457356	60.750627	64.539915
45C	7	49.797383	48.946845	51.019505
45E	1	23.795601	22.794961	25.154487
45E	2	5.4445246	4.8455585	6.4677791
45E	3	13.627534	12.231757	15.39569
45E	4	7.6463315	3.4557572	16.918545
45E	5	36.031278	34.059462	38.3896
45E	6	16.457356	14.750627	18.539915
45E	7	21.797383	20.946845	23.019505
45F	1	5.795601	4.7949605	7.1544866
45F	2	5.4445246	4.8455585	6.4677791
45F	3	13.627534	12.231757	15.39569
45F	4	7.6463315	3.4557572	16.918545
45F	5	12.031278	10.059462	14.3896
45F	6	9.4573557	7.7506273	11.539915
45F	7	2.7973828	1.9468446	4.0195046
45H	1	20.795601	19.794961	22.154487
45H	2	4.4445246	3.8455585	5.4677791
45H	3	19.627534	18.231757	21.39569
45H	4	7.6463315	3.4557572	16.918545
45H	5	36.031278	34.059462	38.3896
45H	6	29.457356	27.750627	31.539915
45H	7	10.797383	9.9468446	12.019505
45J	1	25.795601	24.794961	27.154487
45J	2	14.444525	13.845558	15.467779
45J	3	48.627534	47.231757	50.39569
45J	4	7.6463315	3.4557572	16.918545
45J	5	75.031278	73.059462	77.3896
45J	6	54.457356	52.750627	56.539915
45J	7	23.797383	22.946845	25.019505
45K	1	6.795601	5.7949605	8.1544866
45K	2	3.4445246	2.8455585	4.4677791
45K	3	43.627534	42.231757	45.39569
45K	4	7.6463315	3.4557572	16.918545
45K	5	56.031278	54.059462	58.3896
45K	6	26.457356	24.750627	28.539915
45K	7	3.7973828	2.9468446	5.0195046
45L	1	16.795601	15.794961	18.154487
45L	2	13.444525	12.845558	14.467779
45L	3	20.627534	19.231757	22.39569
45L	4	7.6463315	3.4557572	16.918545
45L	5	62.031278	60.059462	64.3896
45L	6	61.457356	59.750627	63.539915
45L	7	18.797383	17.946845	20.019505
45M	1	11.795601	10.794961	13.154487
45M	2	8.4445246	7.8455585	9.4677791
45M	3	57.627534	56.231757	59.39569
45M	4	7.6463315	3.4557572	16.918545
45M	5	79.031278	77.059462	81.3896
45M	6	43.457356	41.750627	45.539915
45M	7	33.797383	32.946845	35.019505
52A	1	30.795601	29.794961	32.154487
52A	2	15.444525	14.845558	16.467779
52A	3	32.627534	31.231757	34.39569
52A	4	7.6463315	3.4557572	16.918545
52A	5	66.031278	64.059462	68.3896
52A	6	63.457356	61.750627	65.539915
52A	7	12.797383	11.946845	14.019505
52B	1	29.795601	28.794961	31.154487
52B	2	12.444525	11.845558	13.467779
52B	3	49.627534	48.231757	51.39569
52B	4	7.6463315	3.4557572	16.918545
52B	5	72.031278	70.059462	74.3896
52B	6	73.457356	71.750627	75.539915
52B	7	4.7973828	3.9468446	6.0195046
52C	1	30.795601	29.794961	32.154487
52C	2	17.444525	16.845558	18.467779
52C	3	39.627534	38.231757	41.39569
52C	4	7.6463315	3.4557572	16.918545
52C	5	74.031278	72.059462	76.3896
52C	6	67.457356	65.750627	69.539915
52C	7	29.797383	28.946845	31.019505
52E	1	17.795601	16.794961	19.154487
52E	2	15.444525	14.845558	16.467779
52E	3	87.627534	86.231757	89.39569
52E	4	7.6463315	3.4557572	16.918545
52E	5	12.031278	10.059462	14.3896
52E	6	9.4573557	7.7506273	11.539915
52E	7	2.7973828	1.9468446	4.0195046
52F	1	26.795601	25.794961	28.154487
52F	2	10.444525	9.8455585	11.467779
52F	3	62.627534	61.231757	64.39569
52F	4	7.6463315	3.4557572	16.918545
52F	5	87.031278	85.059462	89.3896
52F	6	67.457356	65.750627	69.539915
52F	7	19.797383	18.946845	21.019505
52G	1	33.795601	32.794961	35.154487
52G	2	14.444525	13.845558	15.467779
52G	3	41.627534	40.231757	43.39569
52G	4	7.6463315	3.4557572	16.918545
52G	5	80.031278	78.059462	82.3896
52G	6	76.457356	74.750627	78.539915
52G	7	15.797383	14.946845	17.019505
52H	1	3.795601	2.7949605	5.1544866
52H	2	1.4445246	0.8455585	2.4677791
52H	3	6.6275344	5.2317573	8.3956899
52H	4	7.6463315	3.4557572	16.918545
52H	5	43.031278	41.059462	45.3896
52H	6	54.457356	52.750627	56.539915
52H	7	7.7973828	6.9468446	9.0195046
52I	1	30.795601	29.794961	32.154487
52I	2	27.444525	26.845558	28.467779
52I	3	59.627534	58.231757	61.39569
52I	4	7.6463315	3.4557572	16.918545
52I	5	40.031278	38.059462	42.3896
52I	6	19.457356	17.750627	21.539915
52I	7	17.797383	16.946845	19.019505
52J	1	21.795601	20.794961	23.154487
52J	2	24.444525	23.845558	25.467779
52J	3	92.627534	91.231757	94.39569
52J	4	7.6463315	3.4557572	16.918545
52J	5	102.03128	100.05946	104.3896
52J	6	59.457356	57.750627	61.539915
52J	7	13.797383	12.946845	15.019505
52K	1	42.795601	41.794961	44.154487
52K	2	6.4445246	5.8455585	7.4677791
52K	3	24.627534	23.231757	26.39569
52K	4	7.6463315	3.4557572	16.918545
52K	5	44.031278	42.059462	46.3896
52K	6	48.457356	46.750627	50.539915
52K	7	28.797383	27.946845	30.019505
20A	2	7.8507686	7.401368	8.8033505
20A	3	24.870077	24.010856	26.096435
20A	4	23.620551	22.513092	25.077124
20A	5	16.406076	15.465777	17.704948
20A	6	17.26787	16.515602	18.393525
20A	7	50.567688	49.443306	52.059227
20B	2	12.850769	12.401368	13.803351
20B	3	32.870077	32.010856	34.096435
20B	4	44.620551	43.513092	46.077124
20B	5	13.406076	12.465777	14.704948
20B	6	25.26787	24.515602	26.393525
20B	7	20.567688	19.443306	22.059227
20C	2	16.850769	16.401368	17.803351
20C	3	19.870077	19.010856	21.096435
20C	4	29.620551	28.513092	31.077124
20C	5	60.406076	59.465777	61.704948
20C	6	21.26787	20.515602	22.393525
20C	7	25.567688	24.443306	27.059227
20D	2	26.850769	26.401368	27.803351
20D	3	61.870077	61.010856	63.096435
20D	4	28.620551	27.513092	30.077124
20D	5	43.406076	42.465777	44.704948
20D	6	25.26787	24.515602	26.393525
20D	7	56.567688	55.443306	58.059227
20E	2	5.8507686	5.401368	6.8033505
20E	3	21.870077	21.010856	23.096435
20E	4	23.620551	22.513092	25.077124
20E	5	7.4060756	6.4657769	8.7049475
20E	6	17.26787	16.515602	18.393525
20E	7	11.567688	10.443306	13.059227
20F	2	0.8507686	0.401368	1.8033505
20F	3	5.870077	5.0108561	7.0964352
20F	4	20.620551	19.513092	22.077124
20F	5	16.406076	15.465777	17.704948
20F	6	8.26787	7.5156024	9.3935247
20F	7	7.5676883	6.4433063	9.0592275
20G	2	3.8507686	3.401368	4.8033505
20G	3	8.870077	8.0108561	10.096435
20G	4	14.620551	13.513092	16.077124
20G	5	25.406076	24.465777	26.704948
20G	6	23.26787	22.515602	24.393525
20G	7	11.567688	10.443306	13.059227
20H	2	10.850769	10.401368	11.803351
20H	3	16.870077	16.010856	18.096435
20H	4	30.620551	29.513092	32.077124
20H	5	9.4060756	8.4657769	10.704948
20H	6	14.26787	13.515602	15.393525
20H	7	23.567688	22.443306	25.059227
20I	2	2.8507686	2.401368	3.8033505
20I	3	26.870077	26.010856	28.096435
20I	4	32.620551	31.513092	34.077124
20I	5	13.406076	12.465777	14.704948
20I	6	15.26787	14.515602	16.393525
20I	7	28.567688	27.443306	30.059227
43A	2	7.8507686	7.401368	8.8033505
43A	3	16.870077	16.010856	18.096435
43A	4	32.620551	31.513092	34.077124
43A	5	45.406076	44.465777	46.704948
43A	6	3.26787	2.5156024	4.3935247
43A	7	46.567688	45.443306	48.059227
43B	2	3.8507686	3.401368	4.8033505
43B	3	6.870077	6.0108561	8.0964352
43B	4	8.620551	7.5130915	10.077124
43B	5	17.406076	16.465777	18.704948
43B	6	14.26787	13.515602	15.393525
43B	7	12.567688	11.443306	14.059227
43C	2	0.8507686	0.401368	1.8033505
43C	3	7.870077	7.0108561	9.0964352
43C	4	17.620551	16.513092	19.077124
43C	5	8.4060756	7.4657769	9.7049475
43C	6	6.26787	5.5156024	7.3935247
43C	7	6.5676883	5.4433063	8.0592275
43D	2	3.8507686	3.401368	4.8033505
43D	3	8.870077	8.0108561	10.096435
43D	4	27.620551	26.513092	29.077124
43D	5	17.406076	16.465777	18.704948
43D	6	6.26787	5.5156024	7.3935247
43D	7	64.567688	63.443306	66.059227
43E	2	5.8507686	5.401368	6.8033505
43E	3	19.870077	19.010856	21.096435
43E	4	18.620551	17.513092	20.077124
43E	5	20.406076	19.465777	21.704948
43E	6	8.26787	7.5156024	9.3935247
43E	7	20.567688	19.443306	22.059227
43F	2	6.8507686	6.401368	7.8033505
43F	3	15.870077	15.010856	17.096435
43F	4	17.620551	16.513092	19.077124
43F	5	17.406076	16.465777	18.704948
43F	6	11.26787	10.515602	12.393525
43F	7	40.567688	39.443306	42.059227
43G	2	13.850769	13.401368	14.803351
43G	3	16.870077	16.010856	18.096435
43G	4	38.620551	37.513092	40.077124
43G	5	10.406076	9.4657769	11.704948
43G	6	15.26787	14.515602	16.393525
43G	7	20.567688	19.443306	22.059227
43H	2	5.8507686	5.401368	6.8033505
43H	3	23.870077	23.010856	25.096435
43H	4	28.620551	27.513092	30.077124
43H	5	33.406076	32.465777	34.704948
43H	6	22.26787	21.515602	23.393525
43H	7	40.567688	39.443306	42.059227
43I	2	7.8507686	7.401368	8.8033505
43I	3	5.870077	5.0108561	7.0964352
43I	4	23.620551	22.513092	25.077124
43I	5	15.406076	14.465777	16.704948
43I	6	8.26787	7.5156024	9.3935247
43I	7	32.567688	31.443306	34.059227
Male	1	206.92132	197.17438	219.44819
Male	2	169.4904	160.34674	181.38911
Male	3	608.31739	590.08438	629.87202
Male	4	257.41073	246.42213	271.2457
Male	5	847.01429	824.18092	873.51455
Male	6	618.09991	598.8835	640.70775
Male	7	496.92709	481.24204	515.75648
Female	1	218.92132	209.17438	231.44819
Female	2	216.4904	207.34674	228.38911
Female	3	534.31739	516.08438	555.87202
Female	4	259.41073	248.42213	273.2457
Female	5	745.01429	722.18092	771.51455
Female	6	616.09991	596.8835	638.70775
Female	7	412.92709	397.24204	431.75648
